# *In vitro* activity of cefiderocol against a global collection of carbapenem-resistant *Pseudomonas aeruginosa* with a high level of carbapenemase diversity

**DOI:** 10.1093/jac/dkad396

**Published:** 2023-12-28

**Authors:** Christian M Gill, Debora Santini, David P Nicolau, the ERACE-PA Global Study Group, Elif Aktas, Wadha Alfouzan, Lori Bourassa, Adrian Brink, Carey-Ann D Burnham, Rafael Canton, Yehuda Carmeli, Marco Falcone, Carlos Kiffer, Anna Marchese, Octavio Martinez, Spyros Pournaras, Michael Satlin, Harald Seifert, Abrar K Thabit, Kenneth S Thomson, Maria Virginia Villegas, Julia Wille, Thais Teles Freitas Rezende, Zuhal Cekin, Gulsah Malkocoglu, Desirèe Gijón, Layla Abdullah Tarakmeh, Chun Yat Chu, Christoffel Johannes Opperman, Hafsah Deepa Tootla, Clinton Moodley, Jennifer Coetzee, Sophia Vourli, George Dimopoulos, Dalya M Attallah, Giusy Tiseo, Alessandro Leonildi, Cesira Giordano, Simona Barnini, Francesco Menichetti, Vincenzo Di Pilato, Giulia Codda, Antonio Vena, Daniele Roberto Giacobbe, Lars Westblade, Armando Cardona, Lauren Curtis, Ferric Fang, Gina Thomson

**Affiliations:** Center for Anti-Infective Research & Development, Hartford Hospital, 80 Seymour Street, Hartford 06102, CT, USA; Center for Anti-Infective Research & Development, Hartford Hospital, 80 Seymour Street, Hartford 06102, CT, USA; Center for Anti-Infective Research & Development, Hartford Hospital, 80 Seymour Street, Hartford 06102, CT, USA; Division of Infectious Diseases, Hartford Hospital, Hartford, CT, USA

## Abstract

**Objectives:**

To determine the *in vitro* activity of cefiderocol in a global collection of carbapenem-resistant *Pseudomonas aeruginosa* including >200 carbapenemase-producing isolates.

**Methods:**

Isolates (*n* = 806) from the ERACE-PA Surveillance Program were assessed. Broth microdilution MICs were determined for cefiderocol (iron-depleted CAMHB) and comparators (CAMHB). Susceptibility was interpreted by CLSI and EUCAST breakpoints and reported as percent of isolates. The MIC distribution of cefiderocol in the entire cohort and by carbapenemase status was assessed.

**Results:**

In the entire cohort, cefiderocol was the most active agent (CLSI 98% susceptible; EUCAST 95% susceptible; MIC_50/90_, 0.25/2 mg/L). Amikacin (urinary only breakpoint) was the second most active, with 70% of isolates testing as susceptible. The percentage of isolates susceptible to all other agents was low (<50%) including meropenem/vaborbactam, imipenem/relebactam, piperacillin/tazobactam and levofloxacin. Cefiderocol maintained significant activity against the most commonly encountered carbapenemases including VIM- (CLSI 97% susceptible; EUCAST 92% susceptible) and GES (CLSI 100% susceptible; EUCAST 97% susceptible)-harbouring isolates. The cefiderocol MIC distribution was similar regardless of carbapenemase status, with MIC_50/90_ values of 0.5/4 mg/L, 0.5/2 mg/L and 0.25/1 mg/L for MBL, serine carbapenemase and molecular carbapenemase-negative isolates, respectively.

**Conclusions:**

Cefiderocol displayed potent *in vitro* activity in this global cohort of carbapenem-resistant *P. aeruginosa* including >200 carbapenemase-harbouring isolates. Cefiderocol was highly active against MBL-producing isolates, where treatment options are limited. These data can help guide empirical therapy guidelines based on local prevalence of carbapenemase-producing *P. aeruginosa* or in response to rapid molecular diagnostics.

## Background

Infections caused by carbapenem-resistant *Pseudomonas aeruginosa* remain a significant clinical challenge due to limited treatment options. Carbapenem resistance in *P. aeruginosa* is mediated by several resistance mechanisms including porin/efflux alterations, intrinsic β-lactamases (i.e. cephalosporinase overproduction, OXA-50-like production) and acquired β-lactamases.^1^ Carbapenemase-producing isolates have been well described in different geographical regions including Europe, the Middle East and South America, which represent an even more significant challenge as they are accompanied by cross-resistance to novel β-lactam/β-lactamase inhibitor combinations.^1–3^Although non-carbapenemase mechanisms drive carbapenem resistance in the USA, there is an increasingly recognized threat of carbapenemase-producing *P. aeruginosa* including a multistate outbreak of VIM- and GES-harbouring *P. aeruginosa*.^1,3,4^ Thus, novel agents with activity against carbapenemase-producing isolates are needed to provide viable treatment options.

Cefiderocol, a novel siderophore-conjugated cephalosporin antimicrobial, has been approved for use in the USA and Europe. Cefiderocol is also available in other regions through a novel distribution collaboration with GARDP.^5^ The siderophore moiety of the compound is advantageous in the setting of carbapenem-resistant organisms due to: (i) increased transport into the cell despite decreased membrane permeability; and (ii) stability against carbapenemase degradation including by MBL-producing organisms.^6^ Thus, cefiderocol is a rational option for difficult-to-treat, carbapenem-resistant *P. aeruginosa*, particularly amongst carbapenemase producers, which lack treatment options.

In the era of rapid diagnostic testing, clinicians may have organism identification and detection of resistance determinants, such as carbapenemase genes, prior to traditional phenotypic susceptibility testing.^7^ With actionable data sooner in the clinical course, clinicians will rely on *in vitro* data from isolates with known carbapenemase categorization to support local treatment algorithms in response to rapid molecular diagnostics, particularly when MBLs are detected. The present study assessed the activity of cefiderocol against a cohort of *P. aeruginosa*, including a diverse subset with known carbapenemase positivity.

## Methods

### Isolates

Isolates from the previously described ERACE-PA cohort were assessed. Briefly, isolates were collected from 17 centres in 12 countries from around the globe.^8^ Isolates were acquired if they were determined to be carbapenem resistant by conventional antimicrobial susceptibility testing methods at the submitting site interpreted per their standard breakpoint authority (CLSI or EUCAST).^8^ Isolates were assessed phenotypically and genotypically for carbapenemases as previously described.^8^ A total of 806 isolates were assessed since one isolate previously tested resulted in poor growth.

### Phenotypic profiling

MICs of cefiderocol, amikacin, levofloxacin, meropenem/vaborbactam, meropenem, imipenem/relebactam and imipenem were determined. Cefiderocol MICs were determined using iron-depleted CAMHB, as previously described, per CLSI standards.^9,10^ MICs of the comparator agents were performed on the Thermo Fisher MDRGN2F panels. Due to limited supply caused by a recall, MICs of the comparator agents were completed using broth microdilution plates made in our laboratory as previously described.^8–10^ Routine quality control was conducted per CLSI for all broth microdilution methods and all QCs were in range for each test organism MIC to be valid.^8–10^

### Data analysis

Isolates were classified per susceptibility testing interpretive criteria per CLSI and EUCAST standards.^9,11^ Since meropenem/vaborbactam lacks interpretive criteria for *P. aeruginosa* per CLSI, the meropenem breakpoints were assessed. Due to the limited range of MICs of amikacin on the pre-made Thermo Fisher panels, isolates could only be categorized as ‘S’, ‘I’ and ‘R’ per the CLSI or ‘S’ and ‘R’ per EUCAST for the urinary-only breakpoints for *P. aeruginosa*. For the EUCAST interpretive criteria, the ‘susceptible, increased exposure’ (SIE) breakpoints were assessed for all agents.^11^ Subgroups assessed included categories by genotypic carbapenemase status based on molecular categorization using the Carba-R or Carba-R NxG as previously described.^8^ The MIC distribution and MIC_50/90_ were assessed for cefiderocol in the total population and by genotypic carbapenemase status.

## Results

Cefiderocol was the most active agent in the entire cohort by both CLSI and EUCAST susceptibility criteria, with 98% and 95% of isolates susceptible, respectively. Table 1 describes the percentage of isolates susceptible to each agent tested. Amikacin at the CLSI and EUCAST urinary-only breakpoint was the second most active agent, with 70% of isolates susceptible. Imipenem/relebactam was the second most active β-lactam agent tested in the present study, with 48% of isolates susceptible, although it was less active than ceftolozane/tazobactam (63%) and ceftazidime/avibactam (72%), as previously determined.^8^ Levofloxacin susceptibility was low, showing that resistance to multiple classes was common in the cohort. As expected, since isolates were determined to be carbapenem resistant, as defined by either meropenem or imipenem at the submitting site, <15% of isolates in total were susceptible to either of these agents.

**Table 1.** Percentage of isolates susceptible per CLSI and EUCAST interpretation for all isolates and by carbapenemase classification for cefiderocol

Isolate group, test agent	CLSI susceptible (%)	CLSI intermediate (%)	CLSI resistant (%)	EUCAST susceptible (SIE) (%)	EUCAST resistant (%)
All included isolates, *n* = 806					
* *Cefiderocol	98	<1	<1	95	5
* *Ceftolozane/tazobactam^a^	63	5	32	63	37
* *Ceftazidime/avibactam^a^	72	—	28	72	28
* *Ceftazidime^a^	46	8	46	46	54
* *Cefepime^a^	46	18	36	46	54
* *Meropenem/vaborbactam^b^	17	10	73	42	58
* *Meropenem	14	10	76	38	62
* *Imipenem/relebactam	48	14	37	48	52
* *Imipenem	10	7	83	17	83
* *Piperacillin/tazobactam	28	27	45	28	72
* *Levofloxacin	25	16	59	42	58
* *Amikacin^c^	70	4	26	70	30
Cefiderocol susceptibility by carbapenemase type					
* *VIM, *n* = 136	97	0	3	92	8
* *IMP, *n* = 15	100	0	0	87	13
* *NDM, *n* = 13	85	15	0	69	31
* *GES, *n* = 59	100	0	0	97	3
* *KPC, *n* = 8	100	0	0	100	0
* *Dual carbapenemase,^d^*n* = 12	100	0	0	83	17
* *Phenotypically carbapenemase negative, *n* = 541	99	1	<1	96	4

^a^MIC data reported in reference 8 but presented for comparison.

^b^Meropenem breakpoints used for CLSI.

^c^CLSI urine-only breakpoint.

^d^VIM and KPC, or VIM and IMP, or VIM and OXA-48.

Considering cefiderocol activity against carbapenemase-producing organisms, Table 1 displays the percentage susceptible by carbapenemase category. Amongst VIM- and GES-producing isolates, >90% of isolates were susceptible to cefiderocol regardless of interpretive criteria assessed. Isolates harbouring IMP, KPC and multiple carbapenemases remained highly susceptible to cefiderocol, with >90% and >80% susceptible per CLSI and EUCAST interpretive criteria. NDM-producing organisms had relatively higher MICs compared with the other carbapenemase classes, although 85% and 67% remained susceptible per CLSI and EUCAST criteria. Amongst phenotypically carbapenemase-negative isolates, cefiderocol was highly active, with 99% and 96% susceptible per CLSI and EUCAST, respectively.

**Figure 1.**
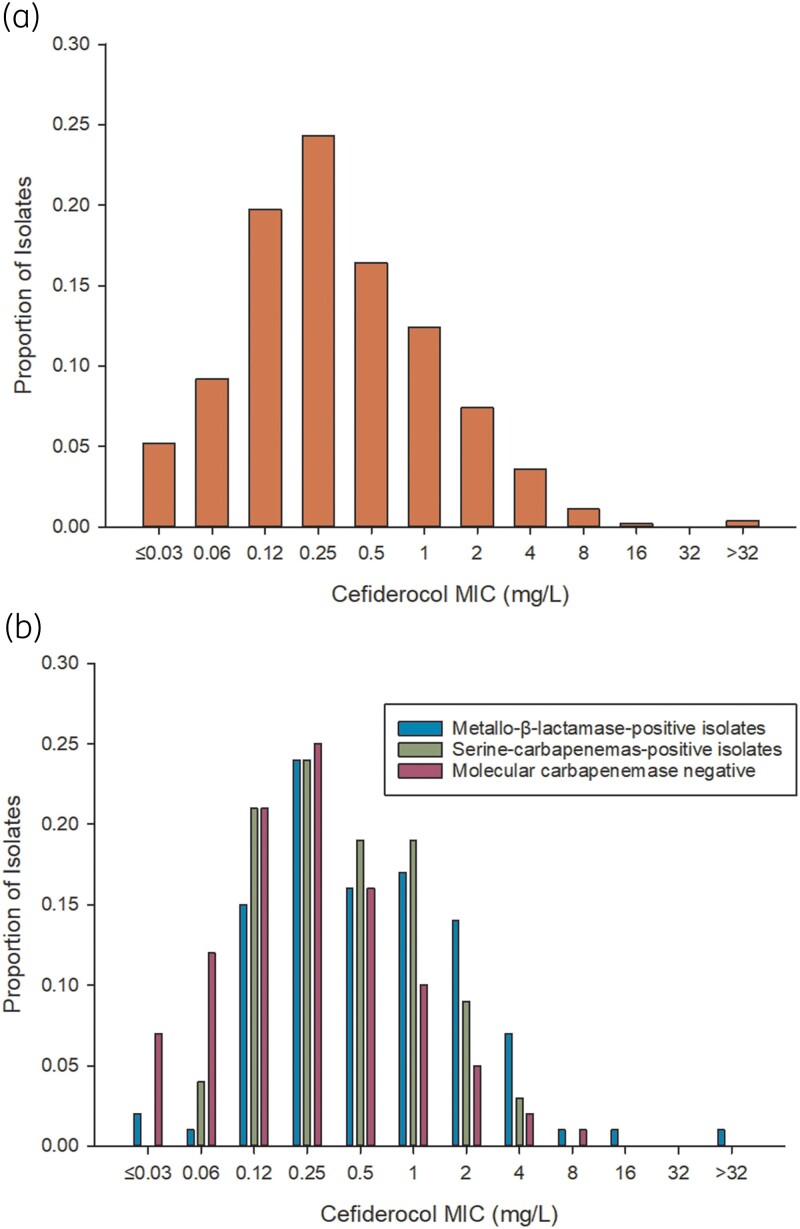
Proportion of isolates by cefiderocol MIC (mg/L). (a) Proportion of isolates by cefiderocol MIC for all included isolates (MIC_50_, 0.25 mg/L; MIC_90_, 2 mg/L; 98% susceptible per CLSI; 95% susceptible per EUCAST). (b) Proportion of isolates by cefiderocol MIC by molecular carbapenemase result (Carba-R NxG). MBL-positive: MIC_50_, 0.5 mg/L; MIC_90_, 4 mg/L; 97% susceptible per CLSI; 89% susceptible per EUCAST. Serine carbapenemase-positive: MIC_50_, 0.5 mg/L; MIC_90_, 2 mg/L; 100% susceptible per CLSI; 97% susceptible per EUCAST. Molecularly carbapenemase negative: MIC_50_, 0.25 mg/L; MIC_90_, 1 mg/L; 99% susceptible per CLSI; 96% susceptible per EUCAST.

Figure 1 describes the MIC distribution for cefiderocol amongst (a) all isolates and (b) by molecular carbapenemase results. The MIC_50_ and MIC_90_ of cefiderocol in the entire cohort were 0.25 and 2 mg/L, respectively. These values were relatively unchanged regardless of molecular carbapenemase detection, with MIC_50/90_ values of 0.5/4 mg/L, 0.5/2 mg/L and 0.25/1 mg/L for MBL-harbouring, serine carbapenemase-harbouring and molecularly carbapenemase-negative isolates, respectively.

## Discussion

With the growing prevalence and diversity of carbapenemase-producing *P. aeruginosa*, safe and efficacious agents are needed to treat these serious infections. These data highlight the *in vitro* potency of cefiderocol in a global collection of *P. aeruginosa* including over 200 isolates with molecularly confirmed carbapenemases. This potency was consistent regardless of carbapenemase status, with >90% and >85% of isolates susceptible per CLSI or EUCAST criteria, respectively, amongst MBL-positive, serine carbapenemase-positive or carbapenemase-negative isolates.

Our findings corroborate those of other surveillance programmes that found a high proportion of meropenem-non-susceptible isolates tested susceptible to cefiderocol *in vitro*. In a global collection from the SIDERO-WT study, the cefiderocol MIC_90_ was 1 mg/L, similar to those seen in our data (2 mg/L).^12^ Although the number of carbapenemase-producing isolates was relatively low, cefiderocol susceptibility was consistently better than other tested agents in the SIDERO-WT programme.^12^ A subsequent analysis specific to isolates from Europe included a larger number of VIM-harbouring isolates (*n* = 76), which found a similarly high proportion of isolates susceptible to cefiderocol with MICs of ≤2 mg/L.^13^ Indeed, relatively higher MICs in NDM-harbouring strains including *Acinetobacter baumannii* and Enterobacterales have been observed, although in the present study focused on *P. aeruginosa*, 85% of isolates had MICs of ≤4 mg/L where the cefiderocol clinical exposure is expected to meet pharmacokinetic/pharmacodynamic targets.^12–14^ The present study adds two important points to the existing literature. Firstly, previously unassessed geographical regions including South America, the Middle East and South Africa were included, adding data in these regions where carbapenemase-producing *P. aeruginosa* are common. Secondly, this cohort includes 136 isolates harbouring VIM, which is the most common carbapenemase detected in *P. aeruginosa* globally. Similarly, 59 isolates harboured GES, an emerging carbapenemase class that has been associated with resistance to our treatments of choice for carbapenem-resistant *P. aeruginosa*, including ceftolozane/tazobactam.^8^

Carbapenemase-producing *P. aeruginosa* have been well described globally, although still considered rare in the USA. Indeed, carbapenemase producing *P. aeruginosa* are commonly encountered in certain regions including Europe, the Middle East and South America, with VIM representing the most common enzymology.^2,3^ Although well recognized globally, numerous outbreaks of VIM-producing *P. aeruginosa* have been reported that have been associated with healthcare transmission and medical tourism.^15,16^ More recently, a multistate outbreak of VIM- and GES-co-harbouring *P. aeruginosa* from contaminated eye drops has been described and has been associated with vison loss and deaths.^4^ Isolates harbouring GES alone are also increasingly recognized amongst *P. aeruginosa* in the USA and abroad.^8,17^ Despite GES positivity conferring significant resistance to ceftolozane/tazobactam, ceftazidime/avibactam and cefiderocol remain highly active *in vitro*, as reported in our cohort. Clinical outcomes data are limited, partially due to the lack of commercially available GES detection capabilities. Rapid molecular diagnostics targeting GES are in development and thus, once commercialized, may offer information to guide therapy selection prior to confirmatory MIC testing.^7,18^

The present study is also useful for developing treatment algorithms based on rapid diagnostic testing. A number of commercially available products can detect the ‘Big Five’ carbapenemases (e.g. VIM, IMP, NDM, KPC and OXA-48) either direct from blood, sputum/bronchoalveolar lavage fluid or culture growth.^7,19^ These assays can provide actionable information sooner in treatment course, even prior to finalized confirmatory susceptibility testing of novel agents.^7,18^ Based on the data presented in this study, cefiderocol is the most likely active agent when MBL-producing *P. aeruginosa* are detected, and thus changing empirical therapy based on these results may decrease the time to active therapy since such enzymes leave IDSA guidance-recommended therapy for difficult-to-treat *P. aeruginosa* (e.g. ceftolozane/tazobactam, ceftazidime/avibactam and imipenem/relebactam) inactive.^20^ These benefits may be enhanced as the molecular detection spectrum expands including GES.^21^

In conclusion, cefiderocol was highly active in this global cohort of *P. aeruginosa* including a diverse collection of carbapenemase-producing isolates. These data confirm the high *in vitro* potency of this agent including amongst MDR isolates that harbour MBLs where active agents are limited. These data are much needed to design treatment algorithms in response to molecular detection of carbapenemase-producing *P. aeruginosa* as their prevalence and the implementation of such technology expands.
